# Spectral Coexistence of QoS-Constrained and IoT Traffic in Satellite Systems

**DOI:** 10.3390/s21144630

**Published:** 2021-07-06

**Authors:** Andrea Munari, Federico Clazzer

**Affiliations:** German Aerospace Center (DLR), Institute of Communications and Navigation, 82234 Weßling, Germany; andrea.munari@dlr.de

**Keywords:** machine-type communications, grant-free access, spectrum-sharing, IoT via satellite

## Abstract

The flourishing of Internet of Things (IoT) applications, characterized by vast transmitter populations and the sporadic transmission of small data units, demands innovative solutions for the sharing of the wireless medium. In this context, satellite connectivity is an important enabler for all scenarios in which terminals are under-served by terrestrial communications and are thus fundamental for providing worldwide coverage. In turn, the design of medium access policies that attain efficient use of the scarce spectrum and can cope with flexible yet unpredictable IoT traffic is of the utmost importance. Starting from these remarks, we investigate in this work the coexistence of a quality of service (QoS)-constrained service with IoT traffic in a shared spectrum as alternative to a more traditional orthogonal allocation among the two services, with an eye on satellite applications. Leaning on analytical tools, we provide achievable rate regions, assuming a slotted ALOHA access method for IoT terminals and accounting for practical aspects, such as the transmission of short packets. Interesting trends emerge, showcasing the benefit of an overlay allocation with respect to segregating the resources for the two services.

## 1. Introduction

Massive machine-type communications (MMTC) and the Internet of Things (IoT) are attracting steadily growing research and industry attention, emerging as a fundamental component for next-generation wireless systems. Driven by a blooming number of IoT applications, this novel communications paradigm aims at serving vast populations of often low-power, low-complexity terminals that generate sporadic traffic in the form of short data packets. Examples of practical relevance span a wide set of scenarios, ranging from smart agriculture or industry, where sensors may collect data (e.g., temperature, pressure, and presence of chemical substances) and deliver status information to a common gateway, to environmental monitoring or asset tracking [1,2,3].

Support for this multitude of use cases is already provided by terrestrial networks in a number of well-established commercial products [4], e.g., LoRa [5,6,7], SigFox [8], Ingenu [9], as well as by standardized approaches, such as NB-IoT and LTE-M [10]. In parallel to this, satellite-based solutions have recently gained traction as a key enabler to provide global coverage to mMTC services [11]. Favored, among other factors, by the significant reduction in launch cost and by the use of off-the-shelves components, such as reprogrammable software defined radios (SDRs), a revived interest toward the deployment of low-Earth orbit (LEO) satellites has characterized the past few years. From this viewpoint, large constellations such as Starlink, OneWeb or the planned Amazon Kuiper are flanked by a growing number of smaller LEO networks focusing on specialized commercial services, e.g., [12,13,14]. Satellite IoT connectivity is one of the key scenarios included in the non-terrestrial networks (NTN) standardization efforts within the 3GPP ecosystem, and is aimed to become part of the standard already from Release 17.

The increasing interest toward mMTC has spurred significant research efforts to tackle a number of challenges that span the whole communications protocol stack. In particular, the intermittent transmission of short packets from IoT terminals calls for innovative approaches. At the physical layer, for instance, the design of the channel codes that operate efficiently over blocks in the order of a few tens to hundreds of bits and with possibly limited channel state information is fundamental [15]. Moreover, the grant-based medium access control policies encountered in traditional human-centric communication systems is largely ineffective for the traffic profile encountered in machine-type applications. As a matter of fact, the overhead needed to coordinate resource sharing in the presence of a massive population of sporadically active transmitters is highly inefficient. In this perspective, a flourishing line of research is emerging, with the proposal of a number of novel *modern* random access protocols [16,17,18,19]. These solutions lean on a joint design of coding and medium access, allowing to achieve spectral efficiencies comparable to those of coordinated schemes under a truly grant-free paradigm, and offer a promising way forward for next-generation mMTC systems. At the same time, random access is already at the core of current IoT communications, as variations of the basic ALOHA strategy [20] are employed by many widely used mMTC solutions [5,8,11].

From this standpoint, grant-free and coordinated access policies offer complementary characteristics, and are commonly employed side by side. Indeed, many communications systems host and provide support to a variety of use cases, ranging from sporadic and often lower-priority mMTC traffic to services with more stringent demands in terms of data rate, reliability and quality of service (QoS). This combination is typically achieved by assigning blocks of orthogonal resources (slices)—in time, frequency, or a combination thereof—to applications with distinct requirements. Relevant examples in this direction are the ETSI S-MIM standard for S-band mobile interactive multimedia [21], focusing on the satellite uplink, or the mobile communications standard 5G from 3GPP [22]. In the former case, the transmission of regular or high data rate traffic is served via demand-assignment procedures, whereas delivery of IoT messages can be attempted on dedicated resource blocks via a variation of spread spectrum ALOHA [23]. Similarly, in 5G, a large amount of the spectrum is dedicated to the enhanced mobile broadband (eMBB) traffic, while IoT messages are sent via the NB-IoT waveform in dedicated sub-bands or unused guard bands [24].

By construction, such approaches avoid interference between different services, easing the provision of proper QoS levels and simplifying waveform design. On the other hand, the reservation of orthogonal sets of resources may not be fully efficient in the presence of machine-type communications. In fact, the unpredictable nature of mMTC traffic inherently leads to significant load fluctuations, hindering the precise tuning of the amount of bandwidth to be allocated and often resulting in an either heavily congested or under-utilized channel. From a different angle, instead, a controlled level of interference may be tolerated by non-mMTC services without violating the target QoS requirements. Indeed, information theoretic results on the multiple access channel show that spectrum sharing and interference cancellation allow to achieve the corner points of the capacity region, suggesting that the coexistence of the two services over a shared band may be beneficial.

Starting from these remarks, we explore in this work the possibility to serve different types of traffic over the same set of resources concurrently. Specifically, we tackle the need to provide uplink support to traffic with a specific QoS target as well as to enable transmission for a best-effort service to IoT devices. Leaning on information theoretic tools, we derive the maximum aggregate rate that can be granted to mMTC traffic without violating the rate and reliability requirements of the coexisting service. We then compare the performance of such an *overlay* configuration to a benchmark setup where resources are orthogonally split, highlighting promising gains.

### 1.1. Related Work

The first research direction considered spectrum coexistence for geographically spaced systems [25,26]. More recently, the idea of sharing bandwidth among non-homogeneous services has started to receive some attention in the context of 5G cellular systems, aiming to go beyond the inefficiencies of orthogonal slicing. In [27], the possibility to multiplex eMBB and ultra-reliable low-latency communications (URLLC) was studied following an information theoretic approach, pinpointing the potential of the idea in a multi-cell cloud radio access network (C-RAN). Additional insights are provided in [28], where the two aforementioned services are flanked by mMTC traffic. Among other scenarios, the work studied a heterogeneous non-orthogonal multiple access solution for the OFDM 5G uplink, where resources are shared between eMBB and mMTC with different reliability requirements. Assuming packets that are long enough to justify an asymptotic information-theoretic analysis, the authors explored via numerical solutions some key trade-offs in terms of rates that can be granted to the distinct services, showing how an overlay allocation can be beneficial in certain regimes. Providing efficient connectivity while guaranteeing the target QoS among several concurrent services was also thoroughly investigated above layer two, e.g., [29]. Finally, a survey on inter-system spectrum sharing focusing on services with equal access rights to the resources can be found in [30].

### 1.2. Main Contributions and Structure of the Paper

Within this line of study, we aim to shed light on the potential of a non-orthogonal resource distribution, as well as to trigger additional research on the topic in a satellite-IoT context. In particular, in our work, we do the following:

- We characterize some fundamental trends for the performance of an overlay allocation of a QoS-constrained service and of mMTC traffic, composed of short packets. Based on practical considerations, we assume the receiver to first attempt decoding the former traffic. In the case of success, its contribution is removed (interference cancellation), and retrieval of the underlying mMTC packets is attempted;
- In this setup, we derive the exact analytical expressions for the maximum aggregate rate that can be granted to mMTC traffic as a function of the requirements in terms of the rate and packet error rate set for the QoS-constrained service;
- Going beyond the approach followed in [28], the analysis relies on information theoretic arguments to capture the impact of short packets transmission, as well as the impact of the length of frames in which the uplink communication is organized.

The remainder of this paper is structured as follows. After introducing the system model in Section 2, we provide in Section 3 the initial insight by comparing the performance of the overlay and orthogonal allocations in a setting characterized by packets long enough to justify the use of asymptotic information theoretic tools, considering both an ergodic and a non-ergodic case. The study is then complemented in Section 4 by exploring the impact of transmission of short packets commonly encountered in mMTC applications, relying on the normal approximation [31,32]. The numerical results are presented and discussed in Section 5, highlighting some fundamental trends and comparing the effectiveness of orthogonal and overlay allocations in all of the considered cases. Finally, Section 6 draws the conclusions, offering some relevant open issues and future research directions.

### 1.3. Notation

We denote random variables (vectors) by uppercase (bold) letters, while we refer to their realizations in lowercase, e.g., *X* and *x*; X and x. The probability mass function (PMF) of a discrete r.v. is denoted as $p_{X}\left(x\right):=P\left\{X=x\right\}$ and that of a discrete random vector as $p_{X}\left(x\right):=P\left\{X=x\right\}$. Moreover, we indicate the conditional PMFs P{X=x|Y=y} as $p_{X}\left(x|y\right)$ and P{X=x|Y=y} as $p_{X}\left(x|y\right)$. Finally, the expectation operator is denoted as E[·], while $I_{k}$ is the identity matrix of size *k*.

## 2. System Model and Preliminaries

Throughout our discussion, we focus on the uplink of a wireless satellite system which supports two distinct services, labeled as $S_{a}$ and $S_{b}$. The former grants specific requirements in terms of data-rate and error probability, and is embodied in our setting by a single user, $s_{a}$, which sends data to the receiver under an average power constraint, $\rho_{a}$ whenever it is granted access to the medium. On the other hand, $S_{b}$ foresees that a large number of terminals share the wireless channel under a slotted ALOHA contention policy [33] to attempt packet delivery in a best-effort fashion, e.g., for mMTC. Specifically, over any time slot allotted to $S_{b}$, a variable number of users *U* access the channel, each transmitting toward the receiver for the whole slot duration with average power $\rho_{b}$. We note that, while the single user for the transmission of a data packet is subject to an average power constraint $\rho_{b}$, the *overall* power injected in the system by service $S_{b}$ varies both with the number of terminals accessing the channel over a single slot and the number of allocated slots. Such a working assumption is representative of the practical implementations of machine-type communications. Following a common approach, *U* is modeled as a Poisson r.v. of parameter λ, independent and identically distributed across slots, so that the following holds:

$$p_{U}\left(u\right)=\frac{\lambda^{u}e^{-\lambda }}{u!}.$$

The assumption is especially representative for traffic generated by a multitude of low duty cycle nodes monitoring and/or reporting data generated by heterogeneous systems and phenomena [34], as often encountered in mMTC satellite systems. Furthermore, we consider for $S_{b}$ no feedback on the outcome of sent packets nor retransmission policies. This approach is in line with the best-effort nature of many IoT applications, where packets losses may be tolerated, awaiting the delivery of a successive update from the monitored devices.

To characterize the performance of the system, we tackle two operation modes, considering an *orthogonal* and an *overlay* allocation for $S_{a}$ and $S_{b}$. In both cases, having in mind the uplink of a satellite system, we assume for the link between a transmitter and the receiver a line-of-sight connection with perfect power control. Accordingly, the channel coefficient at the receiver is a constant and known value for all users, assumed to be unitary for the sake of simplicity, and all users of a given service are received with the same power level.

### 2.1. Orthogonal Allocation

**Figure 1:**
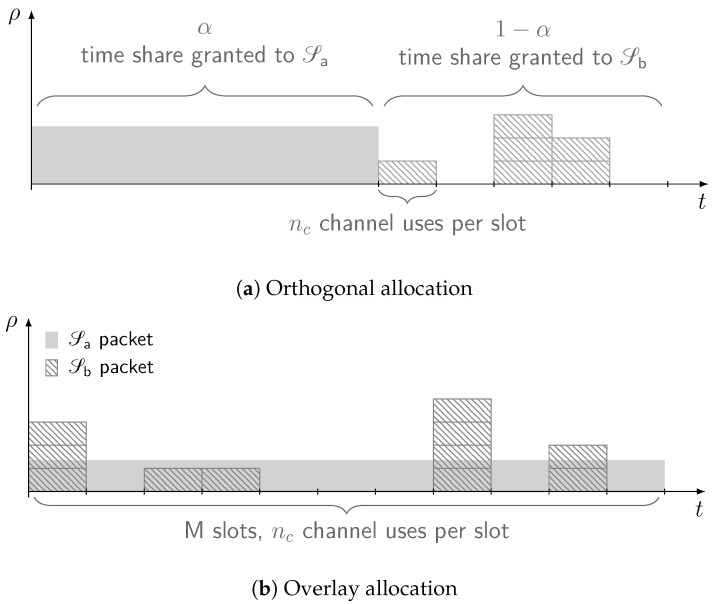
Reference timeline for the orthogonal and overlay resource allocations.

In this configuration, resources are split between services so that no mutual interference between $S_{a}$ and $S_{b}$ arises. Without loss of generality, we assume orthogonality to be achieved in time and focus on the setup exemplified by Figure 1a (the reported analysis and results would hold for an orthogonal allocation in the frequency domain as well). Time is divided into successive frames of equal duration. Within each frame, a fraction α of the time is granted exclusively to $s_{a}$ for data delivery. During this period, the channel output at the receiver over the *ℓ*-th channel use takes the following form:

$$Y_{\ell }=X_{\ell }+W_{\ell }$$

where $X_{\ell }∼CN\left(0,\rho_{a}/\alpha \right)$ is the complex symbol transmitted by the user, and $W_{\ell }∼CN\left(0,\sigma^{2}\right)$ is a zero mean, $\sigma^{2}$ variance, circularly-symmetric complex Gaussian additive noise component. In view of the fraction α of resources available for transmission, the average signal-to-noise ratio (SNR) at the receiver is thus, as follows:

(1) $$\begin{array}{c} \gamma_{a}:=\frac{\rho_{a}}{\alpha \sigma^{2}}. \end{array}$$

As for users of the best-effort service $S_{b}$, channel access is only permitted during the remaining fraction (1−α) of the time. To instantiate a slotted ALOHA policy, the corresponding portion of the frame is assumed to be divided in slots of equal duration, each allowing the transmission of a single packet of $S_{b}$. Denoting by $n_{s}$ the number of channel uses over a slot, the input–output relation over the *ℓ*-th slot conditioned on having U=u users concurrently transmitting can be expressed as follows:

(2) $$Y_{\ell }=\sum_{j=1}^{u}X_{\ell }^{\left(b,j\right)}+W_{\ell }.$$

In (2), $X_{\ell }^{\left(b,j\right)}$ is the codeword transmitted by the *j*-th user of $S_{b}$ active in slot *ℓ*, whose $n_{s}$ components are modeled as i.i.d. circularly-symmetric normal r.v. with zero mean and variance $\rho_{b}$, i.e., $X_{\ell }^{\left(b,j\right)}∼CN\left(0,\rho_{b}I_{n_{s}}\right)$. In turn, $W_{\ell }∼CN\left(0,\sigma^{2}I_{n_{s}}\right)$ is the additive Gaussian noise, leading to the incoming signal vector at the receiver, $Y_{\ell }\in C^{n_{s}}$. Note that when U=0, no user of $S_{b}$ transmits over the slot, and the summation in (2) brings no contribution so that solely the noise component $W_{\ell }$ is observed. As discussed in Section 1, this event, occurring with probability $e^{-\lambda }$, represents a waste of resources, as bandwidth granted to mMTC is not employed to attempt data delivery.

### 2.2. Overlay Allocation

When the system is operated in overlay mode, both services are allowed to access the channel concurrently and without time restrictions, as illustrated in Figure 1b. Such a policy allows $S_{a}$ and $S_{b}$ to enjoy the whole share of resources at the cost of suffering from mutual interference, due to the loss of orthogonality. In this case, the whole frame is assumed to be split in M slots, each consisting of $n_{s}$ channel uses. Using all the resources, $s_{a}$ transmits then a codeword $X^{\left(a\right)}$ of size $n_{s}M$ channel uses. In turn, users of service $S_{b}$ can send a packet over any of the M available slots. To properly capture the signal model, although $s_{a}$ encodes its message across all the available channel uses, it is convenient to express $X^{\left(a\right)}$ as the concatenation of the M sub-codewords transmitted over the slots as follows:

$$X^{\left(a\right)}=\left[X_{1}^{\left(a\right)},\ldots ,X_{M}^{\left(a\right)}\right]$$

where $X_{\ell }^{\left(a\right)}∼CN\left(0,\rho_{a}I_{n_{s}}\right)$, ℓ=1,…,M. Following this notation, the channel output at the receiver over the *ℓ*-th slot conditioned on having U=u users of $S_{b}$ access the channel takes the following form:

(3) $$Y_{\ell }=X_{\ell }^{\left(a\right)}+\sum_{j=1}^{u}X_{\ell }^{\left(b,j\right)}+W_{\ell }.$$

A packet of a best-effort user is thus always affected by the interference coming from $S_{a}$, and possibly by other transmissions of $S_{b}$. Conversely, any sub-codeword sent by $s_{a}$ is received interference-free with probability $e^{-\lambda }$, i.e., if no mMTC user has transmitted over the corresponding slot. It is worth stressing that the number of interfering packets from $S_{b}$ is not known a priori, so the coding rate of $s_{a}$ cannot be dynamically adapted on a slot-by-slot basis.

For both the orthogonal and overlay configurations, we are interested in the maximum sum rate that the system can offer to the best-effort service while granting the QoS requirements of $S_{a}$. Specifically, denoting by $R_{a}$ the rate in bits per channel use of $s_{a}$ and by $R_{b}$ the average aggregate number of bits per channel use decoded at the receiver for $S_{b}$, we aim at deriving the rate region given by all pairs $\left(R_{a},R_{b}\right)$ that allow $s_{a}$ to experience an error probability that is less than or equal to a target value.

## 3. Asymptotic Analysis

In order to gather preliminary insight on the performance of the system, we first consider an asymptotic setting in which the codewords transmitted by $s_{a}$, as well as those by users of $S_{b}$ over a slot, can be made long enough to approach the *classical* information theoretic results.

### 3.1. Orthogonal Allocation

When resources are orthogonally split among services, $s_{a}$ delivers data over an additive white gaussian noise (AWGN) channel with SNR $\gamma_{a}$ defined in (1), yet is allowed to transmit only for a fraction α of the time. In this setting, the user can then communicate with vanishing small error probability for any rate as follows:

(4) $$R_{a}<\alpha \log_{2}\left(1+\gamma_{a}\right).$$

Let us focus instead on the share granted to $S_{b}$, and indicate as $R_{b,\ell }$ the communication rate achieved by the service over the *ℓ*-th slot. If no user accesses the channel (i.e., U=0), then clearly $R_{b,\ell }=0$. Conversely, if a single transmission is performed (U=1, with probability $\lambda e^{-\lambda }$), data are sent over an AWGN channel with capacity as follows:

(5) $$C_{b}=\log_{2}\left(1+\frac{\rho_{b}}{\sigma^{2}}\right).$$

Finally, when more than one user becomes active over the slot, a *collision* takes place, and the channel output at the receiver takes the general form of (2). As will be discussed later (see Remark 1), we assume terminals of $S_{b}$ to encode information at a rate approaching $C_{b}$, as would be done in the absence of interference. Accordingly, in the event of a collision, the actual channel capacity falls below the employed rate, and we regard all packets sent over the slot of interest to be lost. In other words, following a common modeling approach for random access schemes (e.g., [20]), collisions are of a *destructive* nature, and $R_{b,\ell }=0$ whenever more than one user accesses the channel concurrently (U>1). In summary, (i) for U≠1, $R_{b,\ell }=0$, and (ii) for U=1, $R_{b,\ell }=C_{b}$. Combining these observations, the average rate for $S_{b}$ in bits per channel use over a slot readily follows:

(6) $$E\left[R_{b,\ell }\right]=\lambda e^{-\lambda }\;C_{b}.$$

Equation (6) clarifies how, for any given power level $\rho_{b}$, the slotted ALOHA best-effort channel shall be operated at a load λ=1 (pkt/slot) in order to maximize the average aggregate rate. Recalling that only a fraction (1−α) of the resources can be leveraged, the achievable sum-rates for $S_{b}$ are then characterized as follows:

(7) $$R_{b}<\left(1-\alpha \right)\;e^{-1}\cdot \log_{2}\left(1+\frac{\rho_{b}}{\sigma^{2}}\right).$$

**Remark** **1.** *The widely-employed assumption of destructive collisions reflected in (6) is of practical relevance for the considered mMTC setting. Indeed, given that the number of competing terminals over a slot cannot generally be predicted before transmission, a coding scheme that effectively and dynamically takes into account interfering packets may be difficult to develop and goes beyond the complexity of the available terminals. On the other hand, a backoff on the transmission rate to increase resiliency to interference may not be efficient in lightly loaded channels, where sporadic access may lead to having many slots without contention, resulting in significance performance loss for the tight link budget configurations that are typical of mMTC links. Finally, we note that many machine-type applications attempt the delivery of updates that are often repeated or very much correlated in content over time (e.g., reporting of sensed data), positively trading off higher packet loss rates due to interference for a larger aggregate channel sum rate achieved in the absence of coordination among nodes.*

### 3.2. Overlay Allocation

When the system is operated in overlay configuration, the incoming signal at the receiver is the superposition of transmissions performed by both services. Throughout our study, we assume decoding to start from the message sent by $s_{a}$, treating the interference component of $S_{b}$ packets as noise. In the case of successful decoding, ideal interference cancellation is performed, removing the contribution of $s_{a}$ from the overall waveform and presenting to the receiver the M-slot frame populated by packets of $S_{b}$ alone for further processing. Ideal interference cancellation of the packet of user $s_{a}$ can be regarded as a reasonable assumption, especially taking into account that its transmission can enjoy a large number of channel uses, and the data-aided channel estimation can be properly tuned. Moreover, we also note that non-ideal interference cancellation would yield a minor impact on the model by effectively increasing the noise power suffered by the transmissions of service $S_{b}$ since both the signal and the noise are modeled as Gaussian distributed r.vs..

**Remark** **2.** *The assumption on the order of decoding for the supported services stems from two practical considerations. First, as will be further discussed in Section 4 and Section 5, mMTC traffic is typically composed of very short packets—often in the order of a few hundreds of bits— confining pilot symbols to few channel uses per transmitted message in order to avoid excessive overhead. Accurate channel estimation needed for decoding packets of $S_{b}$, especially in the presence of interference from $s_{a}$, may thus not be viable. On the other hand, the longer data units that characterize $S_{a}$ render the cost of stronger pilot sequences manageable, and, together with its more regular and predictable traffic patterns, can lead to estimates of the channel parameters that are good enough to retrieve information and perform accurate interference cancellation. In addition to this, the choice of initiating decoding from $S_{a}$ is driven by QoS arguments. It is indeed reasonable to overlay best-effort mMTC traffic to an existing service only as long as the latter does not experience a significant loss in performance. Along this line of reasoning, the receiver shall be able to decode $S_{a}$ even in the presence of underlying interference, i.e., treating the whole of $S_{b}$ traffic as noise.*

Under this assumption, $s_{a}$ experiences the block-interference channel described in (3), with the interference level remaining constant over a coherence period of one slot duration, and changing to an independent realization during the subsequent $n_{s}$ channel uses. Specifically, conditioned on the number $U_{\ell }$ of users of $S_{b}$ accessing the channel over the *ℓ*-th slot, the r.v. describing the signal-to-interference and noise ratio (SINR) seen at the receiver for $s_{a}$ takes the following form:

$${\mathrm{SINR}}_{\ell }^{\left(a\right)}=\frac{\rho_{a}}{\sigma^{2}+U_{\ell }\;\rho_{b}}.$$

Within this framework, to better grasp the effect of the bursty overlaid mMTC traffic, we first analyze an ergodic setting, later extending our results to the more practically-relevant non-ergodic setup.

#### 3.2.1. Ergodic Case

When the number of slots within a frame is sufficiently large (i.e., M→∞), the codeword of $s_{a}$ experiences, in the limit, all the possible interference values, making it possible to define the ergodic channel capacity.

(8) $$\begin{array}{c} \begin{array}{cc} C_{a}^{\left(e\right)} & =E\left[\log_{2}\left(1+\frac{\rho_{a}}{\sigma^{2}+U\rho_{b}}\right)\right]\\ \; & =\sum_{u=0}^{\infty }\frac{\lambda^{u}\;e^{-\lambda }}{u!}\log_{2}\left(1+\frac{\rho_{a}}{\sigma^{2}+u\rho_{b}}\right). \end{array} \end{array}$$

In this case, a vanishingly small error probability can still be granted to the user for any rate $R_{a}<C_{a}^{\left(e\right)}$. Clearly, from a system design perspective, the overlay operation mode becomes meaningful—and possibly convenient—if the QoS requirements of $S_{a}$ in terms of data rate are met, even in the presence of interference. We are thus interested in configurations for which the achievable rate in overlay matches the one of an orthogonal allocation, i.e., for which, leaning on (4) and (8),

(9) $$\alpha \log_{2}\left(1+\gamma_{a}\right)=\sum_{u=0}^{\infty }\frac{\lambda^{u}\;e^{-\lambda }}{u!}\log_{2}\left(1+\frac{\rho_{a}}{\sigma^{2}+u\rho_{b}}\right).$$

In particular, for any value of α employed to configure a dedicated-resource allocation, (9) imposes a constraint on the tolerable level of interference from $S_{b}$ in overlay mode, limiting the average number of transmissions per slot and thus the intensity of the mMTC traffic. Denoting this value as $\lambda_{m}$, the message of $s_{a}$ is retrieved and its contribution on the incoming waveform is perfectly canceled with probability approaching one for any channel load $\lambda <\lambda_{m}$ of service $S_{b}$. We note that, while a closed-form expression of $\lambda_{m}$ is elusive due to the transcendental nature of (9), its value can easily be determined numerically. If the system is operated satisfying this constraint, the receiver can then process packets sent by mMTC users via slotted ALOHA after having performed interference cancellation, and the average aggregate rate for $S_{b}$ is once again captured by (6). In order to maximize performance while meeting the requirements of the QoS-driven user, $S_{b}$ shall thus be operated at a channel load as follows:

$$\lambda^{*}:=\min\left\{\lambda_{m},1\right\}\;.$$

We recall indeed that values of λ larger than 1 [pkt/slot], even if tolerable by the QoS requirements of $S_{a}$, would be ineffective from a throughput perspective for the mMTC traffic in view of the detrimental effect of destructive collisions.

Combining these remarks, and recalling that both services have now access to the whole set of resources, the rate region $\left(R_{a},R_{b}\right)$ for the ergodic case finally evaluates to the following:

$$\begin{array}{c} \begin{array}{cc} R_{a} & <\sum_{u=0}^{\infty }\frac{\lambda_{m}^{u}\;e^{-\lambda_{m}}}{u!}\log_{2}\left(1+\frac{\rho_{a}}{\sigma^{2}+u\rho_{b}}\right)\\ R_{b} & <\lambda^{*}e^{-\lambda^{*}}\log_{2}\left(1+\frac{\rho_{b}}{\sigma^{2}}\right). \end{array} \end{array}$$

#### 3.2.2. Non-Ergodic Case

Albeit insightful in pinpointing a fundamental trade-off between the achievable rate for $S_{a}$ and the amount of mMTC traffic that can be served, the ergodic setup fails to capture some important aspects of practical relevance. In particular, the assumption of having an asymptotically large number of slots over which user $s_{a}$ can encode its message can become highly inaccurate for typical systems, characterized by frames of a length of few hundreds of slots, e.g., [21]. In such conditions, each codeword of service $S_{a}$ experiences a finite number M of interference realizations, and a vanishingly small error probability can no longer be granted. The QoS requirements of the service are therefore more appropriately characterized by a rate-reliability pair, where $R_{a}$ is complemented by a tolerable error probability for the sent codeword. In the non-ergodic setting, the latter is captured using information theory tools by the outage probability $P_{\mathrm{out}}$. Specifically, for the block-interference channel under study, the two quantities are related as follows:

(10) $$P_{\mathrm{out}}=P\left\{\frac{1}{M}\sum_{\ell =1}^{M}C_{a,\ell }<R_{a}\right\}$$

where $C_{a,\ell }$ denotes the instantaneous capacity for $s_{a}$ over the *ℓ*-th slot, and can be expressed as a function of the number $U_{\ell }$ of $S_{b}$ users transmitting over the slot as follows:

(11) $$C_{a,\ell }:=\log_{2}\left(1+\frac{\rho_{a}}{\sigma^{2}+U_{\ell }\;\rho_{b}}\right).$$

Due to the discrete levels of interference that can be experienced, the r.v. $C_{a,\ell }$ takes values in the alphabet as follows:

$$\begin{array}{c} A=\left\{c_{\ell }\in R\;|\;c_{\ell }=\log_{2}\left(1+\frac{\rho_{a}}{\sigma^{2}+u\rho_{b}}\right),u\in N\right\} \end{array}$$

and its PMF can readily be derived from (11), leading to the following:

(12) $$p_{C_{a,\ell }}\left(c_{\ell }\right)=P\left\{u=\frac{\rho_{a}}{\rho_{b}\left(2^{c_{\ell }}-1\right)}-\frac{\sigma^{2}}{\rho_{b}}\;\right\}=\frac{e^{-\lambda }\lambda^{\beta (c_{\ell })}}{\beta (c_{\ell })!}\;\forall c_{\ell }\in A$$

where the final expression resorts to the ancillary quantity as follows:

$$\begin{array}{c} \beta \left(c_{\ell }\right):=\frac{\rho_{a}}{\rho_{b}\left(2^{c_{\ell }}-1\right)}-\frac{\sigma^{2}}{\rho_{b}}. \end{array}$$

Leaning on this, let us define for compactness the r.v. as follows:

$$V:=\sum_{\ell =1}^{M}C_{a,\ell }.$$

taking values in the set $A_{V}=\left\{v\in R\;|\;\;v=\sum_{\ell =1}^{M}c_{\ell },\;c_{\ell }\in A\right\}$. Observing that the instantaneous capacity values over different slots are i.i.d., the PMF of *V* can simply be computed as the M-fold convolution of (12), to obtain for any $v\in A_{V}$ the following:

$$p_{V}\left(v\right)=\underset{M}{\underbrace{\left(p_{C_{a,\ell }}*p_{C_{a,\ell }}*\cdots *p_{C_{a,\ell }}\right)}}\left(v\right).$$

Accordingly, an exact expression of the outage probability in (10) can finally be derived as follows:

(13) $$P_{\mathrm{out}}=P\left\{V<MR_{a}\right\}=\sum_{\begin{array}{c} v\in A_{V}\\ v<MR_{a} \end{array}}p_{V}\left(v\right).$$

This result offers a useful system design tool. Indeed, for any pair $\left(R_{a},P_{\mathrm{out}}\right)$, (12) and (13) provide an exact characterization of the maximum channel load $\lambda_{m}$ that can be granted to mMTC traffic without violating the QoS requirements of $S_{a}$.

Taking such a constraint into account, let us now focus on $S_{b}$, aiming to derive the aggregate rate $R_{b}$ that can be achieved for the maximum arrival intensity sustainable by $S_{a}$. In this perspective, we observe that two conditions have to be met for an mMTC packet to be retrieved over a slot in a non-ergodic setup: (i) the packet of $s_{a}$ is decoded, and (ii) no other user of $S_{b}$ concurrently accesses the channel over the slot of interest. Note that condition (i) is consistent with the system model assumptions. Indeed, should the message of $s_{a}$ not be decoded, its interference contribution cannot be removed, affecting any underlying mMTC packet. Recalling the discussion of Remark 1, this would prevent the successful retrieval of information for users of $S_{b}$. Furthermore, let us denote by *W* the r.v. taking values in {0,…,M} and counting the number of slots characterized by the transmission of a single user (named also *singleton slots*) of $S_{b}$, which, for the Poisson traffic under study, follows a binomial distribution of parameters M and $\lambda e^{-\lambda }$. Following this notation, for any arrival intensity λ, the aggregate rate of $S_{b}$ can be conveniently expressed as follows:

(14) $$\begin{array}{c} R_{b}=\frac{1}{M}\;\sum_{w=0}^{M}\left[w\log_{2}\left(1+\frac{\rho_{b}}{\sigma^{2}}\right)\cdot \left(1-P\left\{\sum_{\ell =1}^{M}C_{a,\ell }<MR_{a}\;|\;W=w\right\}\right)\right]\cdot p_{W}\left(w\right) \end{array}$$

where, recalling (10), the term within square brackets takes into account how all *w* singleton slots bring a contribution of $\log_{2}\left(1+\rho_{b}/\sigma^{2}\right)$ bits per channel use if, and only if, $s_{a}$ is not in outage. To complete the calculation, we therefore need to derive the outage probability for $S_{a}$ conditioned on the r.v. *W*. To this aim, it is useful to rearrange the conditional expression as follows:

$$\begin{array}{cc} \; & P\left\{\sum_{\ell =1}^{M}C_{a,\ell }<MR_{a}\;|\;W=w\right\}= \end{array}$$

(15) $$\begin{array}{cc} \; & P\left\{w\log_{2}\left(1+\frac{\rho_{a}}{\sigma^{2}+\rho_{b}}\right)+\sum_{\ell ,\;U_{\ell }\neq 1}C_{a,\ell }<R_{a}M\;|\;W=w\right\}= \end{array}$$

(16) $$\begin{array}{cc} \; & P\left\{\sum_{\ell ,\;U_{\ell }\neq 1}C_{a,\ell }<R_{a}M-w\log_{2}\left(1+\frac{\rho_{a}}{\sigma^{2}+\rho_{b}}\right)\;|\;W=w\right\} \end{array}$$

where the first addend on the right-hand side of (15) accounts for the sum of the instantaneous capacity of all *w* slots with a single transmission of service $S_{b}$, whereas the summation takes into consideration the contributions of all other M−w slots (i.e., those with $U_{\ell }\neq 1$). In turn, the PMF of the instantaneous capacity $C_{a,\ell }$ over a *non-singleton* slot can be directly derived from (12), to obtain for any value in the alphabet $\left\{c_{\ell }\in R\;|\;c_{\ell }=\log_{2}\left(1+\frac{\rho_{a}}{\sigma^{2}+u\rho_{b}}\right),\;u\in N\backslash \left\{1\right\}\right\}$,

(17) $$\begin{array}{c} P\left\{C_{a,\ell }=c_{\ell }\;|\;U_{\ell }\neq 1\right\}=\frac{e^{-\lambda }\lambda^{\beta (c_{\ell })}}{\beta (c_{\ell })!}\frac{1}{1-\lambda e^{-\lambda }}. \end{array}$$

where the normalization factor readily follows from the Poisson distribution of the mMTC traffic. Recalling now that also the r.v. $C_{a,\ell }$ over non-singleton slots are i.i.d., the PMF of the sum of M−w such values can be once more derived by taking the (M−w)-fold convolution of (17), allowing to compute (16). Leaning on this result, and plugging the binomial distribution of *W* into (14), $R_{b}$ can be finally computed for any value of λ as follows:

(18) Rb=1Mlog21+ρbσ2∑w=0MwMwλe−λw1−λe−λM−w××1−P∑ℓ,Uℓ≠1Ca,ℓ<RaM−wlog21+ρaσ2+ρb|W=w.

The presented approach provides thus the sought pairs of sustainable rates. As discussed, the maximum sustainable arrival intensity $\lambda_{m}$ for $S_{b}$ can be computed from (13) for any target QoS requirements ($R_{a},P_{\mathrm{out}}$). In turn, mMTC traffic can be operated effectively at any channel load lower than $\lambda^{*}=\min\left\{1,\lambda_{m}\right\}$, with the corresponding aggregate rate $R_{b}$ obtained by evaluating (18) for $\lambda =\lambda^{*}$.

## 4. Finite Blocklength Analysis

Going beyond the asymptotic setting discussed in Section 3, we complement our study by delving into a more practical scenario that closely relates to mMTC applications, where packets transmitted by users can be in the order of few hundreds bits. To capture this aspect we leverage tools of finite-length information theory, and focus in particular on the normal approximation [31,32] to characterize the rates achievable by $S_{a}$ and $S_{b}$.

### 4.1. Orthogonal Allocation

Let us first focus on the orthogonal configuration, and denote by *n* the (finite) number of channel uses available over the whole transmission frame. Following the notation of Section 2, the transmission of $s_{a}$ spans $n_{a}:=\alpha n$ channel uses, and takes place over an AWGN channel of SNR $\gamma_{a}$ defined in (1), i.e., we implicitly focus on values of α such that αn∈N. In this case, a vanishingly small error probability cannot be granted, even in the absence of interference, and the performance of $S_{a}$ is properly described by the couple $\left(R_{a}^{*},P_{e}\right)$, where $R_{a}^{*}$ denotes the maximum rate that can be supported for a codeword error probability $P_{e}$. Specifically, the two quantities are related as follows [31]:

(19) $$R_{a}^{*}\left(P_{e}\right)=C_{a}-\sqrt{\frac{V_{a}}{n_{a}}}\;\;Q^{-1}\left(P_{e}\right)+O\left(\frac{\log n_{a}}{n_{a}}\right)$$

where the channel capacity $C_{a}$ and *channel dispersion*$V_{a}$ are defined by the following expressions:

$$\begin{array}{c} \begin{array}{cc} C_{a} & =\log_{2}\left(1+\gamma_{a}\right)\\ V_{a} & =\frac{\gamma_{a}\left(2+\gamma_{a}\right)}{2{\left(1+\gamma_{a}\right)}^{2}}{\left(\log_{2}e\right)}^{2} \end{array} \end{array}$$

and $Q^{-1}\left(\cdot \right)$ denotes the inverse *Q* function. If we now recall that the user $s_{a}$ transmits only for a fraction α of the time, and approximates (19) by disregarding the terms of order $O\left(\log n_{a}/n_{a}\right)$, the set of rates achievable by $S_{a}$ for a target error probability $P_{e}^{*}$ can be characterized as follows:

(20) $$R_{a}<\alpha \left(C_{a}-\sqrt{\frac{V_{a}}{n_{a}}}\;\;Q^{-1}\left(P_{e}^{*}\right)\right).$$

Consider now service $S_{b}$. In line with what was discussed in Section 3, collisions are once more regarded as destructive, so slots in which more than one user transmits lead to the loss of all the packets sent. In the setup under study, however, a non-vanishing error probability is to be expected for any transmission rate $R_{b}$ even over a *singleton* slot, in view of the finite number $n_{s}$ of available channel uses. Accordingly, introducing the channel dispersion,

$$V_{b}=\frac{\frac{\rho_{b}}{\sigma^{2}}\left(2+\frac{\rho_{b}}{\sigma^{2}}\right)}{2{\left(1+\frac{\rho_{b}}{\sigma^{2}}\right)}^{2}}{\left(\log_{2}e\right)}^{2}.$$

and recalling the channel capacity $C_{b}$ in (5), the maximum rate $R_{b}^{*}$ that can be granted to $S_{b}$ under a codeword error probability ϵ can be approximated as follows:

(21) $$R_{b}^{*}\left(\epsilon \right)≅C_{b}-\sqrt{\frac{V_{b}}{n_{s}}}\;\;Q^{-1}\left(\epsilon \right).$$

In terms of system design, (21) allows then to tune the rate-reliability requirements of $S_{b}$. From this standpoint, we assume within our study mMTC traffic to be of the best-effort nature, i.e., without stringent requirements in terms of reliability, and select the transmission rate in order to maximize the attained spectral efficiency. Specifically, we consider $S_{b}$ to be operated at an error rate $\epsilon^{*}$ that maximizes the information bits per channel use retrieved over a singleton slot:

(22) $$\epsilon^{*}=\underset{\epsilon }{\mathrm{argmax}}\left\{\left(1-\epsilon \right)R_{b}^{*}\left(\epsilon \right)\right\}.$$

Finally, recalling that mMTC traffic can enjoy a share (1−α) of the resources and that the fraction of singleton slots is bounded by $e^{-1}$ (achieved for λ=1), the set of aggregate rates achievable by $S_{b}$ in the orthogonal configuration can be described as follows:

$$\begin{array}{c} R_{b}<\left(1-\alpha \right)e^{-1}\left(1-\epsilon^{*}\right)R_{b}^{*}\left(\epsilon^{*}\right). \end{array}$$

### 4.2. Overlay Allocation

As discussed in Section 2, when the system is operated in overlay mode, the total number of channel uses, *n*, available over a frame is split into M time slots of $n_{s}$ channel uses each, i.e., $n=Mn_{s}$. Let us denote by $U=[U_{1},\ldots ,U_{M}]$ the random vector whose components describe the number of users of $S_{b}$ that transmit over each of the slots. Recalling the i.i.d. Poisson distribution of the r.v. $U_{\ell }$, the joint PMF of U can be expressed for any arrival intensity λ as follows:

(23) $$p_{U}\left(u\right)=\prod_{\ell =1}^{M}\frac{\lambda^{u_{\ell }}e^{-\lambda }}{u_{\ell }!}.$$

Consider now the transmission of $s_{a}$, and condition on a specific realization U=u. In this case, each portion of the sent message experiences a distinct (and independent) interference level, allowing to model the problem as the transmission of an *n* channel uses codeword over M parallel AWGN channels. Following the approach presented in [35] in the context of block-fading, the relation between maximum sustainable rate $R_{a}^{*}$ and codeword error probability $P_{e}$ we can then be approximated as follows:

(24) $$R_{a}^{*}\left(P_{e},u\right)≅\frac{1}{M}C_{a,\mathrm{ov}}\left(u\right)-\sqrt{\frac{V_{a,\mathrm{ov}}\left(u\right)}{n_{s}M^{2}}}Q^{-1}\left(P_{e}\right)$$

where

$$\begin{array}{c} \begin{array}{cc} C_{a,\mathrm{ov}}\left(u\right) & =\sum_{\ell =1}^{M}\log_{2}\left(1+\gamma_{a,\ell }\right)\\ V_{a,\mathrm{ov}}\left(u\right) & =\sum_{\ell =1}^{M}\frac{\gamma_{a,\ell }\left(2+\gamma_{a,\ell }\right)}{2{\left(1+\gamma_{a,\ell }\right)}^{2}}{\left(\log_{2}e\right)}^{2} \end{array} \end{array}$$

and we introduce for convenience the SINR experienced by $s_{a}$ over the *ℓ*-th slot as follows:

$$\begin{array}{c} \gamma_{a,\ell }:=\frac{\rho_{a}}{\sigma^{2}+u_{\ell }\rho_{b}}. \end{array}$$

Solving (24) with respect to $P_{e}$, we can easily obtain the error probability when a given rate $R_{a}$ is employed by $s_{a}$ and a specific realization of the interference pattern U=u is experienced:

(25) $$P_{e}\left(u\right)=Q\left(\frac{\frac{C_{a,\mathrm{ov}}\left(u\right)}{M}-R_{a}}{\sqrt{\frac{V_{a,\mathrm{ov}}\left(u\right)}{n_{s}M^{2}}}}\right).$$

We now observe that, in view of the random nature of the interference of service $S_{b}$, it is more meaningful to evaluate the *average* error probability ${\bar{P}}_{e}$ as follows:

(26) $$\begin{array}{c} {\bar{P}}_{e}=E\left[\;P_{e}\left(u\right)\;\right]=\sum_{u}P_{e}\left(u\right)\;p_{U}\left(u\right) \end{array}$$

by employing (23) and (25). The QoS requirements of $S_{a}$ are properly specified in this case by the pair $\left(R_{a},{\bar{P}}_{e}\right)$.

In this perspective, the result in (26) offers a relevant system design tool. Indeed, for any targeted rate $R_{a}$, an inspection of the equation allows to derive the maximum traffic intensity $\lambda_{m}$ of $S_{b}$ that can be supported without violating the average error probability ${\bar{P}}_{e}$.

Taking the lead from this, let us then focus on $S_{b}$ for which we need to compute the maximum aggregate rate that can be achieved under the constraint on $\lambda_{m}$. In this case, three conditions have to be met for a mMTC message to be retrieved: (i) the message of $s_{a}$ has to be decoded and its interference canceled; (ii) the packet of $S_{b}$ has to be sent over a singleton slot; and (iii) the $n_{s}$-channel use codeword has to be decoded. To account for all these conditions, we follow an approach similar to the one discussed in Section 3.2.2, and condition on the number of singleton slots *W* that take place over the observed frame, writing the aggregate average rate achieved for a traffic intensity λ as follows:

(27) $$\begin{array}{c} R_{b}=\frac{1}{M}\;\sum_{w=0}^{M}w\;R_{b}^{*}\left(\epsilon^{*}\right)\left(1-\epsilon^{*}\right)\left(\sum_{u}\left(1-P_{e}\left(u\right)\right)\;p_{U}\left(u\;|\;w\right)\right)\;p_{W}\left(w\right). \end{array}$$

where we assume that the coding scheme employed by $S_{b}$ has been tuned following (22), so that each of the *w* singleton slots brings a contribution of $R_{b}^{*}\left(\epsilon^{*}\right)\left(1-\epsilon^{*}\right)$ information bits per channel use. Recalling that *W* follows a binomial distribution of parameters $\left(M,\lambda e^{-\lambda }\right)$, the complete evaluation of (27) simply requires to specify the joint PMF of the number of users of $S_{b}$ that transmitted over each of the M slots, conditioned on having W=w singleton ones. The distribution can be computed by considering two cases. First, for any vector u whose number of components with value 1 (i.e., the number of singleton slots in the considered frame realization) is different from *w*, we clearly have $p_{U}\left(u\;|\;w\right)=0$. For any other u, instead.

pU(u|w)=∏ℓ=1Mλuℓe−λuℓ!Mw1−λe−λM−wλe−λw=∏ℓ=1M−wλuℓe−λuℓ!Mw1−λe−λM−w

where the numerator follows from the i.i.d. Poisson distribution of the number of transmissions over a slot, whereas the normalization factor accounts for the probability of having exactly *w* singleton slots out of the available M.

In conclusion, the set of admissible rates for $S_{b}$ can be computed by taking into account the constraint $\lambda_{m}$ imposed by the QoS requirements of $S_{a}$, and by evaluating (27) for all traffic intensities smaller than $\lambda^{*}=\min\left\{1,\lambda_{m}\right\}$.

## 5. Numerical Results

In this section, we present and discuss some numerical results obtained from the general framework developed in Section 3 and Section 4. Bearing in mind the uplink of a satellite IoT system, we target two relevant scenarios. In the former, packets of both service $S_{a}$ and $S_{b}$ reach the receiver with the same power level so that $\rho_{a}/\sigma^{2}=\rho_{b}/\sigma^{2}=0$ dB. This setting reflects the coexistence of two services whose transmitters have a similar hardware (amplifier, antenna) and thus are received with comparable signal strength. In the latter, the QoS-constrained traffic of $S_{a}$ is transmitted with higher power, and we consider the configuration $\rho_{a}/\sigma^{2}=10$ dB, $\rho_{b}/\sigma^{2}=0$ dB. The second setting, instead, assumes that the QoS-constrained service features transmitting units equipped with a better and possibly more costly hardware, thus resulting in a more favorable link budget. The selected configurations are in line with LEO satellite systems targeting IoT applications, e.g., [36].

**Figure 2:**
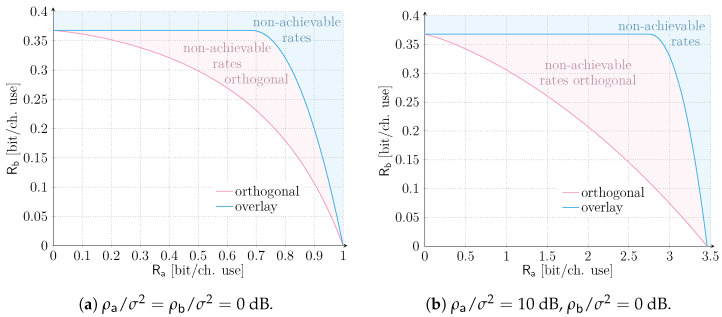
Asymptotic ergodic rate regions for an orthogonal and overlay allocation of resources of services $S_{a}$ and $S_{b}$. Two scenarios are considered: $\rho_{a}/\sigma^{2}=\rho_{b}/\sigma^{2}=0$ dB and $\rho_{a}/\sigma^{2}=10$ dB, $\rho_{b}/\sigma^{2}=0$ dB.

As a starting point, let us focus on the asymptotic, ergodic setting introduced in Section 3. In this case, a vanishingly small error probability can always be granted to $S_{a}$, whose QoS requirements are solely specified in terms of a target data rate $R_{a}$. The corresponding results are reported in Figure 2. The two solid lines represent the boundary of the rate pairs achievable by orthogonal and overlay allocations respectively, according to (4), (7) and (8). The shadowed light-red area comprises rate pairs achievable only with an overlay allocation, whereas the shadowed light-blue area denotes rates that are non-achievable by any of the two schemes. The two SNR scenarios, i.e., $\rho_{a}/\sigma^{2}=\rho_{b}/\sigma^{2}=0$ dB and $\rho_{a}/\sigma^{2}=10$ dB, $\rho_{b}/\sigma^{2}=0$ dB are depicted in Figure 2a,b, respectively. In both configurations, irrespective of the target rate of user $s_{a}$, an overlay allocation is always beneficial to service $S_{b}$ in terms of maximum achievable rates. Incidentally, we note that a slightly different trend was observed in [28], where, even in the asymptotic setting, orthogonal allocation may be beneficial for a small rate region. The discrepancy stems from two main factors. First, different channel models, i.e., AWGN with perfect power control vs. Rayleigh fading, are considered. Second, distinct decoding condition on the mMTC service are assumed. Indeed, while in our case we consider destructive collisions, ref. [28] relies on capture effect and interference cancellation (IC) also for service $S_{b}$ data units.

From Figure 2, we can also infer that, when the two services are operated in overlay, $S_{b}$ can achieve its maximum rate of $e^{-1}≅0.368$ (bit/ch. use) up to $R_{a}\leq 0.68$ (bit/ch. use) for $\rho_{a}/\sigma^{2}=0$ dB, and up to $R_{a}\leq 2.76$ (bit/ch. use) for $\rho_{a}/\sigma^{2}=10$ dB. In other words, increasing the target rate for the QoS-constrained service $S_{a}$ does not impact service $S_{b}$ in this region. Especially for the setting of Figure 2b, there is a very large range of rates for service $S_{a}$, where the IoT traffic is limited by the poor performance of a slotted ALOHA access method. Indeed, the channel code protection of the data unit of user $s_{a}$ could allow a larger channel traffic of service $S_{b}$, beyond λ=1, but cannot be reaped due to the limitation in the medium access. Such a remark hints at how advanced alternatives relying on packet repetition, e.g., [37], can be beneficial to expand the achievable rate region, fostering the need for additional research in this direction. Finally, for $R_{a}>0.68$ (bit/ch. use), or $R_{a}>2.76$ (bit/ch. use) the maximum rate achievable for service $S_{b}$ sees a larger and steeper degradation with respect to the orthogonal allocation as $R_{a}\to \log_{2}\left(1+\rho_{a}/\sigma^{2}\right)$. As a consequence, more caution on the tuning of the traffic intensity for service $S_{b}$ shall be devoted for such rates.

**Figure 3:**
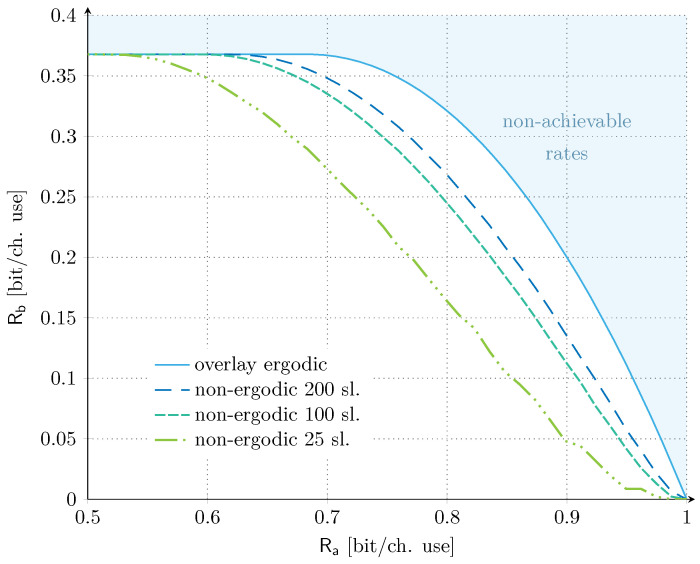
Asymptotic non-ergodic rate regions for overlay allocation. We fix $\rho_{a}/\sigma^{2}=\rho_{b}/\sigma^{2}=0$ dB. The number of time slots are in the set $M\in \left\{25,100,200\right\}$ and the target outage probability for service $S_{a}$ is $P_{\mathrm{out}}^{*}=10^{-3}$. The asymptotic ergodic rate region for the overlay allocation is also provided for reference.

Let us now take a further step and consider the practical constraint imposed by having a finite number of slots within the communications frames (Section 3.2.2). Under these conditions, the transmission of service $S_{a}$ fails to experience all possible interference levels from service $S_{b}$, and a non-ergodic setting has to be considered for the overlay allocation. The corresponding results for a number of time slots in the set $M\in \left\{25,100,200\right\}$ are shown in Figure 3 together with the ergodic benchmark as reference. The set of time slots considered is in line with the literature on random access targeting satellite uplink scenarios, e.g., [37,38]. For our discussion, we set the target (average) codeword error probability for service $S_{a}$ to $10^{-3}$, and focus on the $\rho_{a}/\sigma^{2}=\rho_{b}/\sigma^{2}=0$ dB scenario. Similar trends were also found for the unbalanced SNR scenario, and are not reported here for the sake of compactness. The impact of finite-length frames is clearly visible already for a moderately large number of slots, and the reduction of the achievable rate region becomes even more pronounced as the system operates with smaller values of M. For example, if user $s_{a}$ targets a rate of $R_{a}=0.8$ (bit/ch. use), the maximum achievable rate for service $S_{b}$ is reduced by ∼51% with respect to the ergodic setting, when M=25. The rate $R_{b}$ contraction becomes even more relevant for larger values of $R_{a}$. Conversely, increasing the number of time slots to 100 or 200 mitigates the trend. For the same target rate of $R_{a}=0.8$ (bit/ch. use), the maximum achievable rate for service $S_{b}$ is reduced by only ∼18% with respect to the asymptotic setting, when 200 slots are considered. On the other hand, it is relevant to remark that, also in the non-ergodic setting, there exists a significant range of rate values for service $S_{a}$ that allows to operate $S_{b}$ at the maximum aggregate rate. Such a result confirms the potential of the overlay approach.

**Figure 4:**
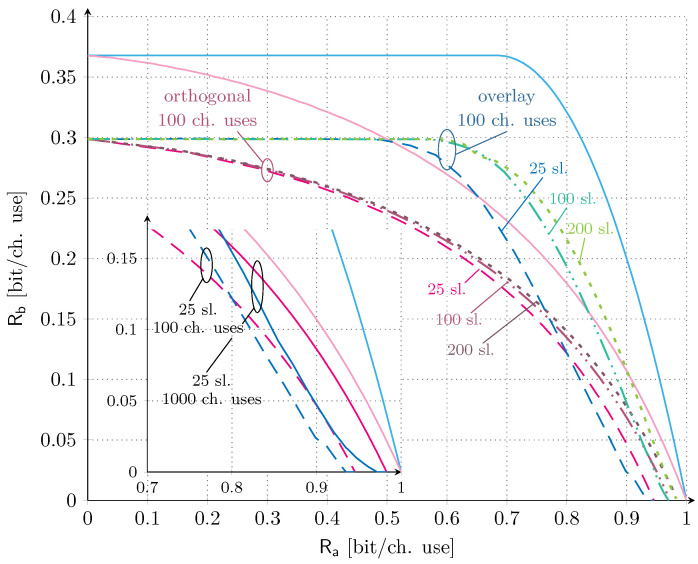
Finite length results for orthogonal and overlay allocations. We fix $\rho_{a}/\sigma^{2}=\rho_{b}/\sigma^{2}=0$ dB. The number of time slots are in the set $M\in \left\{25,100,200\right\}$, the number of channel uses per time slot are $n_{s}\in \left\{100,1000\right\}$ and the target average codeword error probability for service $S_{a}$ is $10^{-3}$. The asymptotic ergodic rate region for the both orthogonal and overlay allocations are also provided for reference.

We also investigate the trends under finite codeword length in Figure 4. In this case, not only the time slots, but also the number of channel uses per time slot are finite and we thus rely on the analysis provided in Section 4. As for the previous scenario, we set the target average codeword error probability of service $S_{a}$ to $10^{-3}$, and consider $\rho_{a}/\sigma^{2}=\rho_{b}/\sigma^{2}=0$ dB. The set of time slots is $M\in \left\{25,100,200\right\}$ and the set of channel uses per time slot $n_{s}\in \left\{100,1000\right\}$. The latter choice is representative of practical mMTC applications, e.g., LoRa [5] and SigFox [8], which enable the transmission of payloads of up to 96 and 2000 bits, respectively. Accordingly, the set of total channel uses in the orthogonal allocation evaluates to $n\in \left\{2500,\text{10,000},\text{20,000},\text{25,000}\right\}$. In the main plot of Figure 4, the orthogonal allocation and the overlay allocation for $M\in \left\{25,100,200\right\}$ and $n_{s}=100$ channel uses per time slot are compared. As we can observe, increasing the number of time slots has a more beneficial impact on the overlay allocation than on the orthogonal one. In the former case, the increase in the number of time slots allows supporting a larger channel load for service $S_{b}$ for the same target outage probability. In the latter case instead, it only impacts the correction term in (20) with respect to capacity, and thus results in a minor benefit on the achievable rate pairs. It is also worth noting that, as expected, the finite number of channel uses strongly affects the maximum achievable rate of service $S_{b}$, reducing it by ∼18% with respect to the asymptotic case. More interestingly, in contrast with what was discussed in the asymptotic setting, a region where an orthogonal allocation is superior to the overlay configuration emerges. Focusing on the M=25 case, such a region is well highlighted in the subplot, where both scenarios with $n_{s}\in \left\{100,1000\right\}$ are depicted. Although rather limited and only for rates of service $S_{a}$ larger than 0.8 (bit/ch. use), such inversion suggests that reserving a portion of time for the IoT service alone is beneficial instead of letting the two services compete completely.

**Figure 5:**
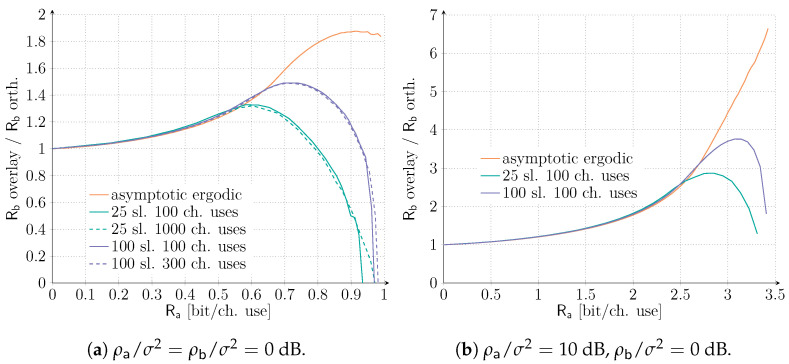
Ratio of the maximum rate achievable by service $S_{b}$ with an overlay allocation ($R_{b}$ overlay) over the one with an orthogonal allocation ($R_{b}$ orth.) as a function of $R_{a}$. Both the ergodic asymptotic setting and the finite length scenarios are presented. In the latter, we set the target average codeword error probability of service $S_{a}$ to 10^−3^ for both SNR setups. M = 25 and M = 100 slots together with *n_s_* = 100 and *n_s_* = 300 channel uses are investigated for $\rho_{a}/\sigma^{2}=\rho_{b}/\sigma^{2}=0$ dB, while only *n_s_* = 100 channel uses for $\rho_{a}/\sigma^{2}=10\mathrm{dB},\rho_{b}/\sigma^{2}=0\mathrm{dB}$ are shown.

In order to further investigate this aspect, we provide in Figure 5a different angle on the considered results. In particular, we analyze the ratio of the maximum rate achievable by service $S_{b}$ with an overlay allocation to the one obtained in the orthogonal case as a function of $R_{a}$. Values larger than 1 thus identify regions of $R_{a}$ where an overlay allocation can outperform the orthogonal one, so that a more aggregate mMTC rate is achieved while granting the same performance to the QoS-constrained service. To highlight the impact of short-packet transmissions, we compare the asymptotic, ergodic setting with finite length scenarios. In the latter case, we set the target average codeword error probability of service $S_{a}$ to $10^{-3}$. Let us focus first on the setting $\rho_{a}/\sigma^{2}=\rho_{b}/\sigma^{2}=0$ dB. As already shown in Figure 2, under the asymptotic, ergodic setting, the overlay allocation is always beneficial. However, Figure 5a reveals the presence of an optimal operating point at $R_{a}≅0.92$ (bit/ch. use) for which the benefit achieved by the overlay allocation is maximized. A similar trend can also be observed for both M=100 and M=25, although for progressively smaller values of rate $R_{a}$. Moreover, the improvement reduces as well with the number of available time slots, going from a peak 87.5% increase in rate $R_{b}$ for the overlay allocation with respect to the orthogonal one in the asymptotic setting, to a 48.7% improvement for M=100 and 32.7% for M=25. The plot also confirms that, in practical finite-length setups, there exist values of $R_{a}$ for which an orthogonal allocation is convenient (i.e., the ratio falls below 1). Nonetheless, such a region drastically reduces by increasing the number of slots available over a frame. Finally, the effect of the number of channel uses appears to have a minor impact. Taking the lead from this, we explore in Figure 5b the configuration $\rho_{a}/\sigma^{2}=10$ dB, $\rho_{b}/\sigma^{2}=0$ dB, focusing only on $n_{s}=100$. Increasing the SNR enjoyed by the QoS-constrained service drastically increases the advantage perceived by the IoT traffic when an overlay allocation is adopted. Rate exceeding a six-fold improvement for the IoT traffic can be achieved in the asymptotic ergodic setting, while, even for as few as 25 slots, more than a two-fold increase in $R_{b}$ is expected when in overlay allocation. Interestingly, the region where the orthogonal allocation is superior for the equal SNR scenario appears to vanish in this scenario.

## 6. Conclusions and Outlook

In this paper, we investigated the potential of letting two services, a QoS-constrained ($S_{a}$) and a mMTC ($S_{b}$), share a common spectrum by overlaying their transmissions in an AWGN scenario modeling the uplink of a satellite communication system. The receiver attempts decoding of $S_{a}$ and, if successful, removes its contribution by means of IC, possibly allowing to retrieve data units of the mMTC traffic transmitted with a slotted ALOHA policy. Leveraging analytical tools, we have shown that an overlay allocation is beneficial in most situations, compared to a more traditional orthogonal allocation among the two services. Starting with an asymptotic scenario, where both the codewords and the number of time slots are very large, we delved into a non-ergodic setting (finite number of time slots) and the more practical finite-length regime (both time slots and codewords are finite). Achievable rate regions and expected gains for the IoT aggregate rate when the overlay allocation is adopted are presented for all scenarios. Furthermore, a rate tuple $\left(R_{a},R_{b}\right)$ maximizing the improvement on the aggregate rate for the mMTC service is identified, showcasing a possible optimal operating point for the overlay system.

The presented work aims at stimulating further research in the context of the spectral coexistence between mMTC and QoS-constrained traffic in satellite scenarios. The possibility to upgrade the medium access policy of the mMTC service to more advanced solutions and exploring the benefits of *modern* random access schemes is an interesting direction. For example, the use of repetition-based solutions, e.g., [37], may unleash further benefits of the overlay allocation for low enough rates of the QoS-constrained service. In particular, when strong forward error correction is adopted on service $S_{a}$, the mMTC service is not able to fully exploit it since high channel load values are detrimental in slotted ALOHA (cf. Figure 2 and Figure 4, for example). A repetition-based scheme instead, can reap the rewards of a stronger interference rejection in $S_{a}$ (lower rate) by heavily loading the physical layer with packet copies. Furthermore, we focused in this paper on perfect power control, i.e., all terminals of service $S_{b}$ are received with the same power. In practical scenarios, in turn, fading and topology trigger variability in the received power levels so that the *capture* of packets is viable at the receiver. The impact of this aspect is also worth exploring, as it may trigger other relevant benefits and trade-offs, all the more so when coupled with IC, becoming especially relevant in the context of modern random access policies for IoT. The decoding order of services—first $S_{a}$, then IC and subsequently $S_{b}$—can also be further investigated when power variability is present, along the lines of [28]. Finally, the applicability of the overlay allocation shall be investigated in a real-world scenario, entailing details such as the link budget, topology of the transmitters and effective error correcting code, among others.

## Data Availability

Not applicable.
